# Meta-Analysis: Diagnostic Accuracy of Anti-Cyclic Citrullinated Peptide Antibody for Juvenile Idiopathic Arthritis

**DOI:** 10.1155/2015/915276

**Published:** 2015-02-19

**Authors:** Yan Wang, Fengyan Pei, Xingjuan Wang, Zhiyu Sun, Chengjin Hu, Hengli Dou

**Affiliations:** ^1^Department of Laboratory Medicine, General Hospital of Jinan Military Area, Jinan, Shandong 250031, China; ^2^Department of Laboratory Medicine, Jinan Central Hospital, Jinan, Shandong 250013, China; ^3^Division of Chest Disease, The Fourth Hospital of Jinan, Jinan, Shandong 250031, China

## Abstract

*Objective*. To estimate the diagnostic accuracy of the anti-CCP test in JIA and to evaluate factors associated with higher accuracy. *Methods*. Two investigators performed an extensive search of the literature published between January 2000 and January 2014. The included articles were assessed by the Quality Assessment of Diagnostic Accuracy Studies tool. The meta-analysis was performed using a summary ROC (SROC) curve and a bivariate random-effect model to estimate sensitivity and specificity across studies. *Results*. The bivariate meta-analysis yielded a pooled sensitivity and specificity of 10% (95% confidence interval (CI): 6.0%–15.0%) and 99.0% (95% CI: 98.0%–100.0%). The area under the SROC curve was 0.96. Sensitivity estimates were highly heterogeneous, which was partially explained by the higher sensitivity in the rheumatoid factor-positive polyarthritis (RF+ PA) subtype (48.0%; 95% CI: 31.0%–65.0%) than in the other subtypes (17.0%; 95% CI: 14.0%–20.0%) and the higher sensitivity of the Inova assay (17.0%; 95% CI: 14.0%–20.%%) than the other assays (0.05%; 95% CI: 2.0%–11.0%). *Conclusions*. Anti-CCP antibody test has a high specificity for the diagnosis of JIA. The sensitivity of this test is low and varies across populations but is higher in RF+ PA than in other JIA subtypes.

## 1. Introduction

Juvenile idiopathic arthritis (JIA) is the most common chronic rheumatic disease of obscure etiology in children and adolescents younger than 16 years; the worldwide prevalence of JIA is approximately 0.07 to 4.01 per 1000 children [1]. Although JIA may be transient and self-limiting, up to 10% of affected children remain severely disabled [2–4]. According to the International League Against Rheumatism (ILAR) criteria, JIA, as an umbrella term, was further divided into different subgroups, which include rheumatoid factor- (RF-) negative polyarthritis (RF− PA), RF-positive polyarthritis (RF+ PA), systemic-onset arthritis (SA), oligoarthritis (OA), psoriatic arthritis (PsA), enthesitis-related arthritis (ERA), and undifferentiated arthritis (UA) [5]. Each subtype exhibits distinct genetic, immunologic, and clinical characteristics [6]. Furthermore, with new and effective therapeutic approaches becoming widely available, such as biological agents, JIA subtypes also differ in terms of therapeutic response and prognosis [1, 7]. Therefore, early identification of flu-like symptoms is important for optimal patient management and prevention of joint destruction.

The diagnosis of JIA is based primarily on clinical manifestations. There are only a few serological markers with confirmed value in JIA. Of these, IgM RF is the most well-characterized autoantibody and is included in the ILAR criteria for IgM RF+ PA JIA [8–10]. IgM RF is found in about 40%–50% of patients with the PA subtype of JIA. The presence of IgM RF is thought to be correlated with erosions and radiographic progression. However, there are different opinions concerning its role in the diagnosis and prognosis of the PA subtype of JIA. As a nonspecific marker of JIA, IgM RF can be also found in other diseases and in healthy people. Recently, various circulating non-RF antibodies have been discovered and found to be of potential diagnostic and clinical value. However, most of these autoantibodies, including antinuclear (ANA), antikeratin (AKA), anti-perinuclear factor (APF), and anti-RA33 antibodies, also show limited value in supporting decisions regarding the clinical course and therapy of patients with JIA [5, 8, 11–14].

In recent years, some studies have elucidated a role for antibodies against cyclic citrullinated peptide (CCP), which is a synthetic peptide mimicking the relevant epitopes of filaggrin. Anti-CCP antibodies are of outstanding diagnostic and prognostic value in rheumatoid arthritis (RA) and are now included in the revised diagnostic criteria for RA [15–19]. Anti-CCP antibodies can also be detected in the sera of patients with JIA. Several studies have reported that anti-CCP antibodies are an important indicator of destructive disease in JIA as well as in RA [13, 20–25]. Unfortunately, anti-CCP assays have inconsistent accuracy, with reported sensitivity rates ranging from 1.8% to 71.4% in mainly JIA patients with RF+ PA or patients with joint damage, whereas the specificity usually exceeds 95% [6, 13, 20, 22–39] (Figure 3). This variability is probably attributable to the different proportions of the various JIA subtypes in the studies as well as variations in the ethnic background of the patients or in the commercially manufactured assays used in the studies. Thus, the clinical significance of anti-CCP antibodies in JIA remains unclear [40].

Worldwide comparative data on anti-CCP antibody tests have not yet been critically synthesized, and the effect of prevalence on the accuracy of the test has not been reviewed. The aim of this diagnostic meta-analysis is to evaluate and summarize the available evidence on the diagnostic accuracy of anti-CCP assays in children with JIA. In addition to estimating the overall accuracy, we evaluated the quality of the included studies and explored factors that may be responsible for heterogeneity among the studies.

## 2. Methods

### 2.1. Data Sources and Searches

We developed a protocol for the review and conformed to standard reporting guidelines for the systematic review of diagnostic studies [41, 42]. We searched two electronic databases, PubMed (January 2000 to May 2014) and EMBASE (January 1980 to May 2014) for studies published in English that evaluated the diagnostic accuracy of the anti-CCP assay for the diagnosis of JIA.

Our searches were based on combinations of the following index terms: anti-CCP antibody, anti-CCP antibodies, anti-cyclic citrullinated peptide antibodies, anti-CCP antibody, anti-cyclic citrullinated peptide antibody, juvenile chronic arthritis, juvenile rheumatoid arthritis, juvenile chronic arthritis, JIA, JRA, diagnostic accuracy, sensitivity, specificity, and so on. Abstracts of relevant studies were reviewed, and appropriate articles were then retrieved. Reference lists of the included studies were scrutinized to catch missed references.

### 2.2. Study Selection

Two authors (Yan Wang and Hengli Dou) independently evaluated titles or abstracts matching the inclusion criteria. The analysis was based on the following inclusion data:

1. observational studies without intervention imposed by researchers,
2. studies that evaluated the diagnostic accuracy of anti-CCP antibodies in JIA,
3. studies that enrolled at least 10 JIA patients and at least 10 control persons,
4. studies that provided enough data to allow calculation of sensitivity and specificity for the diagnosis of JIA.

The diagnostic reference standard of JIA was the 2004 ILAR criteria [43] or the 1987 American College of Rheumatology (ACR) criteria [42]. Reviews, conference abstracts, and letters to editors were excluded.

### 2.3. Data Extraction and Study Quality Assessment

Two researchers (Xingjuan Wang and Zhiyu Sun) independently assessed eligible studies. The researchers were blinded to the publication details, and discrepancies were resolved by consensus. Data retrieved from the reports included author, publication year, disease duration, proportion of female participants, number of participants, control group composition, method of testing for anti-CCP antibodies, and anti-CCP cutoff value. We also listed the diagnostic value, in terms of diagnoses missed (false-negative) and misdiagnoses (false-positive), of anti-CCP antibodies.

Two researchers (Yan Wang and Fengyan Pei) independently evaluated the methodological quality of each study by using 14 standard items from the Quality Assessment of Diagnostic Accuracy Studies (QUADAS) score to identify study bias and limitations [41].

### 2.4. Statistical Analysis

We extracted data from the primary studies to obtain the four cell values of a diagnostic two-by-two table and recalculated sensitivity, specificity, positive likelihood ratio (PLR), negative likelihood ratio (NLR), and diagnostic odds ratio (DOR) with 95% confidence intervals (CIs) for each study. We also estimated heterogeneity by means of the Cochran *Q* method, the test of inconsistency (*I* ^2^), and forest plots. In order to combine data and estimate the underlying relationship between specificity and sensitivity, a summary receiver operating characteristic (SROC) curve was constructed to summarize true-positive rates (TPR = sensitivity) and false-positive rates (FPR = 1 − specificity) [44]. Data were analyzed using Meta-Disc, a software for statistical analysis (version 1.4; Ramony Cajal Hospital, Madrid, Spain) [45].

To assess the diagnostic accuracy of anti-CCP assays in JIA, we performed a random-effects bivariate regression analysis, which takes this correlation into account, and reported pooled accuracies with 95% CIs [46]. We also generated hierarchical summary receiver operating characteristic (HSROC) curves to summarize the global test performance. HSROC curves differ from traditional ROC curves in allowing accuracy to vary by each individual study and represent summary plots of the sensitivity and specificity, with 95% joint intervals in two-dimensional space. They provide information on the overall performance of a test across different thresholds. The closer the curve is to the upper left-hand corner of the plot (sensitivity and specificity are both 100%), the better the performance of the test [47]. For bivariate random-effects regression and HSROC analyses, we used Stata 12.0 (Stata Corporation, College Station, TX, USA) [48]. Exploring the possible reasons for heterogeneity between studies is an important aspect of conducting a meta-analysis. If necessary, subgroup analysis was to be conducted according to the JIA subtype, commercial brand of anti-CCP assay, and components of the control group in order to analyze the sources of heterogeneity among the studies. The Spearman correlation coefficient of sensitivity and 1 − specificity was calculated to assess the threshold effect. Finally, funnel plots were used to explore potential publication bias in our meta-analysis [49].

## 3. Results

### 3.1. Search Results

**Figure 1:**
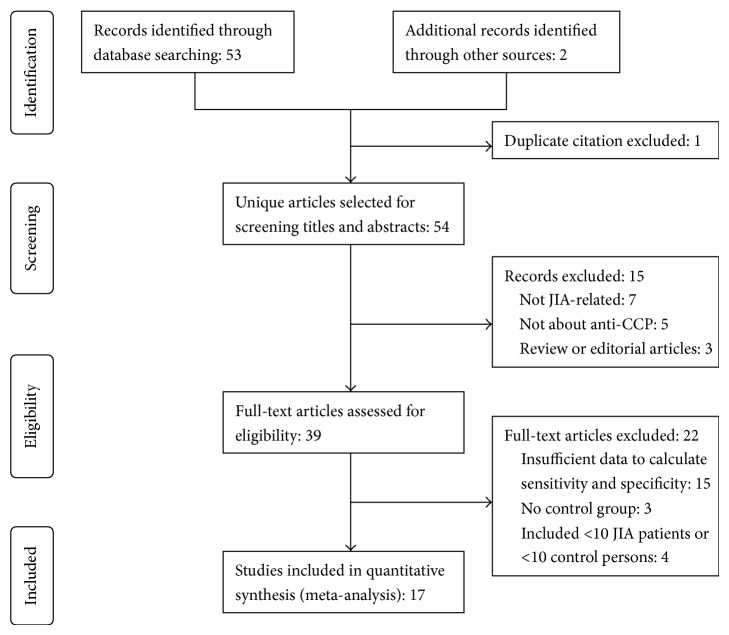
Articles selection process and reasons for exclusion of studies.

A total of 53 records were identified through database searching with additional two citations identified by manual review of the bibliographic material from review articles and included articles (Figure 1). After removing one duplicate study, the titles and abstracts for 54 records were screened for eligibility. Of these, 39 records were identified as being potentially relevant, and their full-text articles were retrieved for a more thorough review. After excluding 22 records based on the data in the full-text article, the remaining 17 studies enrolling 1868 patients met the inclusion criteria and were included in the meta-analysis.

### 3.2. Characteristics of Studies

In 17 included studies, one was prospective [32] and sixteen were retrospective in design [6, 13, 20, 22, 24–26, 28, 30, 31, 33–35, 37–39].  Table 1 summarizes the characteristics of the included articles. The median number of JIA patients was 95, and their median age was 11 years. The median proportion of female patients was 66%, and the median duration of illness was 3.7 years. In 11 studies, a second generation or anti-CCP2 test was used, and anti-CCP3 and anti-CCP1 tests were used in four and two studies, respectively. Of the 17 studies, 8 (47.1%) used a commercial assay manufactured by Inova (San Diego, California, USA) (cutoff, 20 U/mL), 4 used an assay produced by Euroimmun (Luebeck, Germany) (cutoff, 5 or 40 RU/mL), and 5 (29.4%) used assays produced by other manufacturers (cutoff, 50 or 70 AU/mL). The characteristics of the control groups varied among the 17 articles. Five studies used healthy persons as a control group. Eight studies used a mix of healthy volunteers and patients with other diseases, while four studies used patients with other diseases as controls.

### 3.3. Study Quality

**Figure 2:**
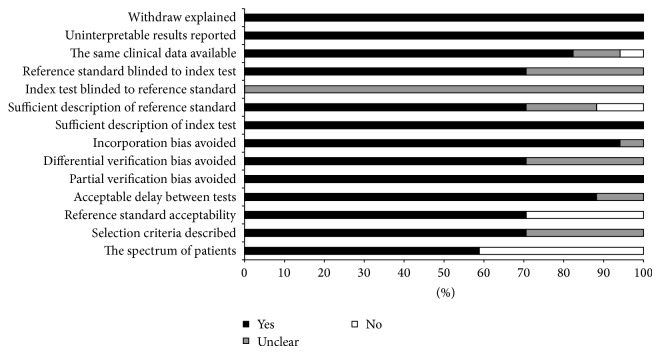
Assessment of the 17 included studies quality with use of the Quality Assessment of Diagnostic Accuracy Studies (QUADAS) tool.

**Figure 3:**
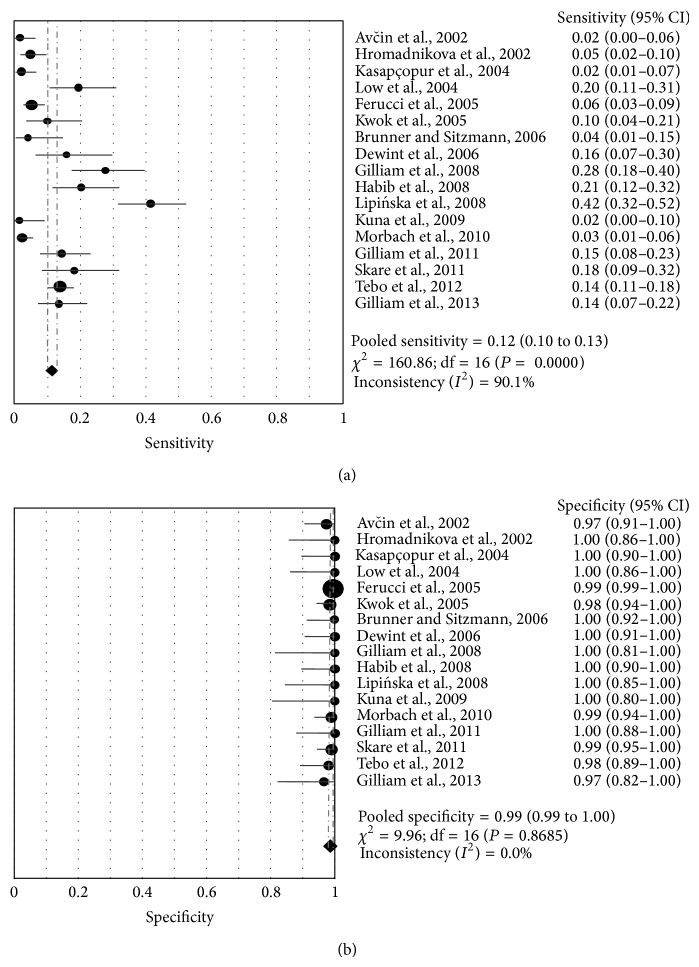
Sensitivity (a) and specificity (b) plots for anti-CCP antibody test in the diagnosis of JIA. Only the first author of each study is given. The circles represent the sensitivity and specificity of one study and the black line its confidence interval. The diamond at the bottom of each plot is the pooled sensitivity or specificity value.

Figure 2 displays the proportion of studies that accomplished each QUADAS criterion. The median score for quality was 12. Of the 17 studies, 6 (35%) met 13 criteria, 5 met 12 criteria, 2 met 11 criteria, and only 4 studies met less than 10 criteria. Regarding study design and execution, all studies were identified as retrospective research. In addition, all studies adequately described the technical approach of assaying anti-CCP antibodies. However, they did not definitively report whether the assessors of the anti-CCP assay results were blinded to the reference standard. Four studies used the 1987 ACR criteria, and eight studies used the 2001 ILAR criteria as the reference standard for JIA. Both criteria were accepted as eligible reference standards. All studies clearly describe the definition of the anti-CCP assay implemented and patient selection criteria used. All of the studies explained patient withdrawals from the study and reported uninterpretable or intermediate test results. All studies enrolled patients with known JIA, and enrollment was retrospective. Characteristics of these patients were fully described in 82% of the studies.

### 3.4. Results of All Included Studies

Figure 2 shows a forest plot of the sensitivity, specificity, and 95% CI in the 17 studies included in the present meta-analysis. Specificity seemed to be more consistent across the studies than sensitivity, with sensitivity estimates ranging from 1.8% to 41.7% and specificity estimates ranging from 97.3% to 100%. In comparison with the univariate analysis, a bivariate analysis for sensitivity achieved a similar estimate. Bivariate pooled sensitivity and specificity estimates for the anti-CCP assay were 10% (95% CI: 6.0%–15.0%) and 99% (95% CI: 98.0%–100%), respectively (Table 2). The PLR for anti-CCP antibody testing was high enough for this assay to be used as a rule-in test (PLR: 10.05; 95% CI: 3.59–30.07), while the NLR was not sufficiently low for the assay to be used as a rule-out test (NLR: 0.91; 95% CI: 0.87–0.96). The area under the SROC curve was 0.96 (95% CI: 0.96–0.99), indicating a moderate and perfect level of overall accuracy.

**Figure 4:**
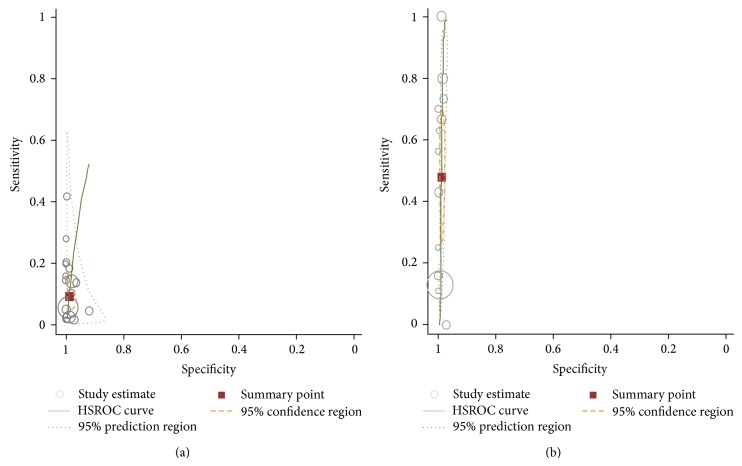
Hierarchical summary receiver operating characteristic (HSROC) curves of anti-CCP antibody assay for the diagnosis of JIA among all included studies (a) and RF+ PA subtype subgroup (b), together with the summary receiver operating characteristic (SROC) curve (solid line) and the bivariate summary estimate (solid square), with the corresponding 95% prediction ellipse (outer dotted line) and 95% confidence ellipse (inner dashed line). The symbol size for each study is proportional to the study size. The bivariate analysis presents studies as sensitivity and specificity data, which are identified by the coordinates of corresponding points in sensitivity-specificity space. Ellipses indicate the bidimensional limits of confidence of sensitivity and specificity for each diagnostic criterion: solid line, hsROC curve; square, bivariate summary estimate; dashed line, 95% confidence area; dotted line, 95% prediction area.

Figure 4(a) shows the HSROC curve with the 95% confidence region and 95% prediction region. The HSROC curve also showed greater variation in sensitivity than in specificity. The 95% confidence region was narrow, improving the precision of the studies in the pooled estimate. The 95% prediction region (amount of variation between studies) was also wide, suggesting heterogeneity between the studies.

### 3.5. Subgroup Analysis for Investigation of Heterogeneity

The Spearman correlation coefficient for anti-CCP was 0.015, indicating that heterogeneity was not caused by a threshold effect. Therefore, subgroup analyses were conducted to investigate heterogeneity in sensitivity and, to a lesser degree, in specificity. We performed subgroup analysis by restricting studies according to JIA subtypes, control group types, and commercial brands of anti-CCP assay. Table 2 summarizes the pooled accuracy measures for the whole included studies and the subgroups using the bivariate random-effects regression method. There were not enough studies to use meta-regression as a strategy to identify predictors of test accuracy and explain inconsistency in results across studies.

Analysis of the subgroups according to JIA subtype clearly showed a high degree of variability in sensitivity estimates, whereas specificities in all subgroups were similar. In the subgroups classified by JIA subtypes, the pooled sensitivity of anti-CCP assay was highest in the RF+ PA subgroup (48%, 95% CI: 0.31–0.65), followed by that in the SA subgroup (0.23%, 95% CI: 0.00–0.20) and that in the RF− PA subgroup (0.06%, 95% CI: 0.03–0.11). In the RF+ PA subgroup, the overall PLR, NLR, and AUC were 53.27 (95% CI: 27.78–102.16), 0.53 (95% CI: 0.38–0.72), and 0.99, respectively (Table 2). The HSROC curve is shown in Figure 4(b). The RF− PA and OA subgroups had lower sensitivity (6% and 2%, resp.) than the RF+ PA subgroup. Specificity was consistent among the subgroups. Anti-CCP assay performed better in patients with the RF+ PA subtype of JIA.

In the analysis of the subgroups classified by commercial manufacturer, the Inova subgroup had a higher sensitivity (0.17, 95% CI: 0.14–0.20) than that obtained in the case of other manufacturers (0.05, 95% CI: 0.02–0.11). However, the Inova subgroup did not display a better trade-off relationship between sensitivity and specificity, the AUC of which was 0.80 (*Q* ^*^ = 0.49). Moreover, this result should be interpreted with caution because of the limited data available for the other manufacturers.

Analysis of the subgroups according to type of control group showed that the type of control group (healthy controls or patients with other rheumatic diseases) had no noticeable effect on pooled accuracy estimates. There was no evidence of publication bias in the overall or subgroup analyses.

## 4. Discussion

JIA is a complex autoimmune disease [50] and may result in both short- and long-term disability, such as joint damage and deformity, growth abnormalities, and osteoporosis with fragility fractures, as well as persistent arthritis into adulthood [9, 51, 52]. Therefore, early diagnosis and effective treatment are crucial for preventing irreversible structural complications in JIA and increasing quality of life [53].

According to the ILAR criteria, the diagnosis of JIA depends primarily on clinical manifestations after the exclusion of infections and other inflammatory diseases and lacks reliable serological support [8]. It is difficult to establish the diagnosis of JIA, especially in the early stage of the disease, since the clinical symptoms are often not characteristic [24, 54–56]. Currently, none of the serological markers for JIA, such as IgM RF, APF, and AKA, appears to be useful for the diagnosis and assessment of the disease course. Therefore, novel suitable serological markers for JIA are urgently needed.

Anti-CCP antibodies are now considered to have outstanding diagnostic and prognostic value in assessing progressive radiological damage in adult RA, with sensitivities of 65%–80% and specificities of 89%–100% [9, 54, 56–58]. Moreover, positivity for anti-CCP antibodies has been included in the 2010 revised ACR criteria for RA. Since 2002, several studies have assessed the diagnostic efficacy of anti-CCP antibodies in JIA, and opinions about their value in children are inconsistent [6, 13, 20, 22, 24–39, 43]. Substantial differences are present in the reported occurrence rates of anti-CCP in JIA patients, with results varying from 1.8% to 41.7% [6, 13, 20, 22, 24–39, 43]. In short, most studies have suggested that anti-CCP can be detected in JIA patients at low levels and less commonly than in adults with RA.

To date, there has been no comprehensive systematic review evaluating the diagnostic accuracy of anti-CCP antibody assays in JIA. In the present analysis, we identified that anti-CCP antibodies can be detected in JIA patients, but these antibodies generally have high specificity, with low and highly variable sensitivity. These findings are consistent with those of most previous studies, as summarized in our meta-analysis [6, 13, 20, 22, 25, 28, 29, 31–35, 38]. Because the pooled sensitivity (12%) was very low for anti-CCP antibody assay, the good diagnostic accuracy of this assay was mainly due to its perfect specificity (99%). Positive results of the anti-CCP antibody test can rule in (PLR, 5.95) the diagnosis of JIA. The DOR is a single indicator of test performance, with higher values indicating better discriminatory test performance. In our meta-analysis, the DOR of all included studies was 11.0 (95% CI: 3.53–34.23), demonstrating that anti-CCP tests could be useful in the diagnosis of JIA. Therefore, in the presence of a positive anti-CCP antibody test result in a child with specific clinical symptoms, a clinician can confidently distinguish JIA from other early undifferentiated arthritides in children and take appropriate measures, such as biologic, anti-inflammatory, and antirheumatic therapy [59–61]. However, a negative test does not mean the absence of JIA and should be confirmed by other diagnostic laboratory tests or clinical manifestations. Unlike RF or ANA antibodies, which are established markers of JIA, anti-CCP antibodies are rarely detected in healthy controls and non-JIA or non-RA patients. Hence, the anti-CCP assay has better specificity than other laboratory measures and facilitates the differential diagnosis of early undifferentiated arthritis in children.

To investigate the possible source of heterogeneity among the included studies, stratified analysis was applied step by step. Because JIA includes seven subtypes, we thought that it was necessary to analyze our data for each disease subtype, even though this resulted in small numbers and large confidence intervals for some of the analyses.

Fourteen studies evaluated anti-CCP levels in different JIA subtypes [6, 13, 20, 22, 24, 25, 28, 30, 31, 33–35, 37, 39]. The ILAR JIA subtypes in the included studies were RF+ PA, RF− PA, SA, and OA. We did not determine the diagnostic accuracy for other JIA subtypes because of insufficient data. Similar to previous reports [20, 22, 24, 25, 28, 31, 33, 34], the disease subtype subgroup analysis showed that the diagnostic value of anti-CCP antibodies was best in the RF+ PA subtype, with the highest sensitivity (48%) and specificity (99%). Anti-CCP antibodies can also be found incidentally in several other JIA subtypes, but they are commonly present in the RF+ PA subtype. Among the different JIA subtypes, the long-term prognosis for children is best in the case of the OA subtype and worst in the case of the RF+ PA subtype, which is severe and represents the pediatric form of RA [62]. Not surprisingly, anti-CCP antibodies have been shown to be more prevalent in this particular subtype of JIA, although in a smaller proportion of patients than that reported in RA [29]. The markedly increased PLR (101.45) indicated that positivity for anti-CCP improved the probability of true positivity in the diagnosis of RF+ PA, which means that patients who are positive for this antibody are more likely to develop RA. Considering the above results, we conclude that anti-CCP antibodies could be a marker of RF+ PA and indicate an increased potential of progression to joint destruction. In addition to its diagnostic utility, the anti-CCP antibody has been evaluated as a predictor of future JIA development.

The subgroup analysis for different manufacturers showed that the Inova anti-CCP assay had slightly higher sensitivity (17.0%) than that obtained using assays from other manufacturers (5.0%). However, this result should be interpreted with caution because of the marked heterogeneity between manufacturers and the inadequate data for other manufacturers. Three generations of anti-CCP assays are currently available commercially. However, a subgroup analysis for different generations of anti-CCP assays could not be performed due to the limited data available. Earlier reports have found that the prevalence of anti-CCP in JIA is low, ranging from 0% to 19% [6, 13, 22, 24–26, 30, 39]. However, the most recent study has reported a high prevalence rate, ranging from 21% to 42% with different assays [20, 28, 31]. Our results further confirmed that anti-CCP2 and anti-CCP3 tests have higher sensitivity for the diagnosis of rheumatic diseases, such as RA or JIA, than the anti-CCP1 test. However, we caution readers that our meta-analysis is based on a small number of studies and that there is still considerable uncertainty regarding the usefulness or nonusefulness of these tests.

JIA is a heterogeneous disease whose precise etiology remains unknown but is thought to involve an autoimmune process [24, 63]. The pathogenetic and pathological effects of anti-CCP antibodies in JIA remain unclear. Citrulline is generated by posttranslational enzymatic deimination (citrullination) of arginine residues [64]. Hromadnikova et al. suggested that autoreactive T and B cells induced by a previous infection (memory cells) can be reactivated by the later occurrence of citrullinated epitopes in the stressed synovium [39]. This discovery led to the development of assays, including anti-CCP assays to measure antibodies recognizing citrullinated antigens as a diagnostic test for RA; this eventually led to the development of the first commercial enzyme-linked immunosorbent assay (ELISA) for anti-CCP [61, 65]. Since the anti-CCP ELISA was introduced in 2000, three generations of anti-CCP tests have been developed and are currently available commercially [61].

The low sensitivity of the anti-CCP test in JIA in general and moderate sensitivity in RF+ PA subtype shows that anti-CCP selects for a specific subgroup of JIA patients but is not valuable for the diagnosis of JIA in general. This may be explained by the fact that JIA is a heterogeneous group of disorders and no unified approach of anti-CCP tests. RF+ PA disease has a disease course very similar to RA development and may be the childhood counterpart of adult RA [66]. Actually, anti-CCP antibodies in RF+ PA have been shown to have higher sensitivity than the other subtypes in JIA, although in a smaller proportion of patients than that reported in adults with RA [21]. A relationship between anti-CCP and erosive joint disease has been shown by some researchers [9, 57, 60, 61]. Further prospective studies are needed to determine the presence of these antibodies at an early stage in the RF+ PA subset which might be useful in identifying patients who are at a higher risk for the development of joint erosions. Its low sensitivity does not allow its use as a screening test, but because of its high specificity, it may become one of the most useful serological tests for the diagnosis of JIA, especially RF+ PA subset.

The strengths of our meta-analysis include the use of a standard protocol, a comprehensive literature search strategy, quality assessment with QUADAS, and the involvement of two independent reviewers in all stages of the review process. Moreover, we used rigorous methods of data analysis, including bivariate random-effects regression models and HSROC curve analyses. To our knowledge, this is the first meta-analysis of the diagnostic value of the anti-CCP assay in JIA patients.

In addition to these strengths, the present study has several limitations that should be considered. First, the major limitation of our findings is the relatively small number of available studies and many of the included studies had a small size (Table 1) [26, 30, 32, 35]. The small number of studies involved in subgroup analysis might have induced heterogeneity in sensitivity and specificity in between studies and potentially limits the generalisability of our results. Second, a majority of the included papers were retrospective studies and have incomplete follow-up data. In comparison with prospective studies, retrospective studies may increase selection biases and the potential problem of dealing with a lot of missing data. Therefore, the time course for the detection of anti-CCP antibodies and the development of erosions could not be determined. Third, as expected, considerable heterogeneity was found in the pooled estimates. Despite using subgroup analysis, a large proportion of the heterogeneity remained unexplained. Many factors, possibly contributing to this residual heterogeneity, could not be assessed because they were only reported in few studies. For example, joint erosions in patients with JIA are likely to have an important effect on anti-CCP test performance. This information was mentioned in only 17% of the included studies [6, 13, 28]. Most studies were cross-sectional in design and therefore did not address the relationship of anti-CCP antibodies with treatment, disease activity, and long-term outcomes. Fourth, studies also had methodological limitations. In particular, most studies using the QUADAS tool did not state whether the assessors of the anti-CCP assay results were blinded to the reference standard. Unblinded assessment could lead to an overestimation of the test performance. Fifth, because of the linguistic abilities of our research team, we limited our search to papers written in English; we missed papers written in other languages and this might have led to a language bias. Sixth, we may have missed some eligible gray literature, such as conference abstracts and letters to the editors, because we included only diagnostic studies that provided sufficient information on sensitivity and specificity. Seventh, in eligible studies authors used anti-CCP tests of different generations (anti-CCP1, anti-CCP2, and anti-CCP3), from different manufacturers (Inova, Euroimmun, Axis-Shield (Dundee, United Kingdom), Human GmbH (Mainz, Germany), and Euro-Diagnostica (Arnhem, Netherlands)), with different cutoff values. To some extent, we only performed a qualitative analysis and not a quantitative analysis.

Further observational prospective studies should be recommended to assess the prevalence of anti-CCP antibodies prior to or at the onset of JIA, especially in RF+ PA patients. Additionally, longer observation will provide a definitive answer as to whether the anti-CCP antibody titer changes over time and the relationship between anti-CCP and erosion formation, the course of the disease, long-term prognosis, and therapy. Additionally, further prospective evaluation is warranted to determine the prevalence, significance, and predictive value of specific autoantibodies to citrullinated proteins/peptides (i.e., anti-citrullinated type II collagen antibodies, anti-citrullinated vimentin, and others) and elucidate their role in JIA in general and in different subtypes of the disease [34, 38]. Most importantly, it is critical for ongoing development of international reference standards to harmonize anti-CCP test results, so as to make the interpretation of these results consistent and help clinicians improve the diagnosis and treatment of JIA patients. We believe that a number of important questions should be solved in future works in order to reduce heterogeneity in studies.

In conclusion, despite the limitations mentioned above, the current evidence suggests that anti-CCP antibodies are not as common in JIA as in RA. However, this antibody assay is a very valuable tool for the diagnosis of the RF+ PA subtype of JIA, and a possible predictor of the progression of JIA, with high specificity and moderate sensitivity for the diagnosis of this subtype of JIA.

The anti-CCP assay should be used as a screening method in daily laboratory practice for the initial diagnosis of patients with suspected JIA, especially in children with polyarthritic disease. In order to decrease the misdiagnosis rate, a combination of anti-CCP antibodies, other laboratory tests, and clinical manifestations may be necessary in clinical practice.

## Figures and Tables

**Table 1:** Characteristics and test performance of the included studies of autoantibodies against cyclic citrullinated peptide.

First author, year (reference)	Location	Reference standard	Test^⋇^	Assay manufacturer^#^	Cutoff value	Mean or median age, y	Women %	Mean duration of illness, y	Case number	Control participants	Result			
											TP	FP	FN	TN
Avčin, 2002 [13]	Italy	NA	CCP1	Euro-Diag-nostica	70 AU/mL	8.7	72.48	3.6	109	Healthy children (*n* = 30) SLE (*n* = 45)	2	2	107	73

Hromadnikova, 2002 [39]	Czech Republic	NA	CCP1	Euro-Diag-nostica	50 U/mL	16.5	54.29	1	140	Healthy children (*n* = 24)	7	0	133	24

Kasapçopur, 2004 [25]	Turkey	ILAR	CCP2	Euroimmun	40 IU/mL	8.8	59.0	4.3	122	Healthy children (*n* = 15) SLE (*n* = 19)	3	0	119	34

Low, 2004 [24]	United States	ACR	CCP2	Inova	NA	11.6	NA	12.4	66	Healthy controls (*n* = 25)	13	0	53	25

Ferucci, 2005 [6]	United States	ACR	CCP2	Axis-Shield	5 U/mL	14.69	73.91	9.66	230	Healthy children development of type 1 diabetes (*n* = 688)	13	4	217	684

Kwok, 2005 [22]	China	ILAR	CCP2	Inova	20 AU/mL	15.0	40.7	4.3	59	Other rheumatic diseases (*n* = 68) Healthy volunteers (*n* = 60)	6	2	53	126

Brunner, 2006 [26]	Austria	NA	CCP2	Euroimmun	5 U/mL	11.0	66.67	2.1	45	Healthy newborns (*n* = 42) Other autoimmunopathies patients (*n* = 38) Patients with other underlying diseases (*n* = 34)	2	9	43	105

Dewint, 2006 [30]	Belgium	ILAR	CCP2	Imunoscan	25 U/mL	10.5	78.3	1.3	44	Nonpolyarticular JIA (*n* = 38)	7	0	37	38

Gilliam, 2008 [20]	United States	ACR	CCP2	Inova	20 U/L	13.39	82.4	5.38	68	Healthy children (*n* = 18)	19	0	49	18

Habib, 2008 [28]	Egypt	ILAR	CCP2	Inova	20 U/mL	10.6	44.12	3.7	68	JSLE (*n* = 14) Healthy children (*n* = 20)	14	0	54	34

Lipińska, 2008 [31]	Poland	ILAR	CCP2	Euroimmun	5 RU/mL	NA	61.46	NA	96	Children with functional cardiovascular system dysfunction (*n* = 22)	40	0	56	22

Kuna, 2009 [32]	Croatia	ILAR	CCP2	Euroimmun	5 RU/mL	11	73	3.4	56	Other juvenile rheumatic diseases (*n* = 17)	1	0	55	17

Morbach, 2010 [33]	Germany	NA	CCP2	Human GmbH	NA	NA	NA	NA	191	Healthy children (*n* = 88)	5	1	186	87

Gilliam, 2011 [34]	United States	ACR	CCP3	Inova	20 U/mL	11.2	80.2	3.9	96	Pediatric-onset SLE (*n* = 19) Healthy children (*n* = 10)	14	0	82	29

Skare, 2011 [35]	Brazil	ILAR	CCP3	Inova	20 U/mL	25.3	73.4	15.31	49	Healthy volunteers (*n* = 100)	9	1	40	99

Tebo, 2012 [37]	United States	ILAR	CCP3	Inova	20 U/mL	6.5	65.5	NA	334	Healthy children (*n* = 50)	47	1	287	49

Gilliam, 2013 [38]	United States	NA	CCP3	Inova	20 U/mL	12	81.1	NA	95	SLE (*n* = 19) Healthy children (*n* = 10)	13	1	82	28

^*^CCP = cyclic citrullinated peptide; SLE = systemic lupus erythematosus; ILAR = International League of Associations for Rheumatology; ACR = the American College of Rheumatology criteria; JSLE = juvenile systemic lupus erythematosus; JIA = juvenile idiopathic arthritis; TP = true-positive; FP = false-positive; FN = false-negative; TN = true-negative; NA = not available.

^⋇^The generation of anti-CCP test in the following: methods section of the articles referenced. CCP3 refers to CCP3 from Inova and not CCP3.1.

^#^The locations of the assay manufactures are as Euro-Diagnostica (Arnhem, Netherlands), Euroimmun (Luebeck, Germany), Inova (San Diego, California, USA), Axis-Shield (Dundee, United Kingdom), and Human GmbH (Mainz, Germany).

**Table 2:** Summary of subgroup analysis of the included studies by different study characteristics.

Variables	Number of studies	SN (95% CI)	SP (95% CI)	+LR (95% CI)	−LR (95% CI)	DOR (95% CI)	AUC
All studies	17	0.10 (0.06–0.15)	0.99 (0.98–1.00)	10.05 (3.59–30.07)	0.91 (0.87–0.96)	11.00 (3.53–34.23)	0.96
Subtype of patients							
RF+ PA	14	0.48 (0.31–0.65)	0.99 (0.98–1.00)	53.27 (27.78–102.16)	0.53 (0.38–0.73)	101.45 (43.74–235.28)	0.99
RF− PA	11	0.06 (0.03–0.11)	0.99 (0.98–1.00)	5.26 (1.86–14.92)	0.95 (0.91–0.99)	5.53 (1.89–16.19)	0.99
SA	12	0.23 (0.00–0.20)	0.99 (0.98–1.00)	2.71 (0.28–26.40)	0.99 (0.93–1.04)	2.76 (0.27–28.25)	0.97
OA	13	0.02 (0.08–0.62)	0.99 (0.98–1.00)	2.65 (0.81–8.78)	0.99 (0.96–1.01)	2.70 (0.80–9.10)	0.67
ERA	7	—	—	—	—	—	—
PsA	3	—	—	—	—	—	—
UA	2	—	—	—	—	—	—
Control group type							
ORD	10	0.07 (0.04–0.14)	0.98 (0.94–0.99)	4.04 (1.05–15.53)	0.94 (0.89–0.99)	4.28 (1.06–17.26)	0.99
HC	13	0.09 (0.06–0.15)	0.99 (0.98–1.00)	15.51 (4.62–52.07)	0.91 (0.87–0.96)	17.00 (4.94–58.52)	0.99
ORD and HC	7	0.09 (0.05–0.16)	0.99 (0.97–1.00)	7.59 (2.20–26.20)	0.92 (0.87–0.98)	8.22 (2.27–29.73)	0.99
Generation of anti-CCP tests							
CCP 1	2	—	—	—	—	—	—
CCP 2	11	0.10 (0.05–0.18)	0.99 (0.99-1.00)	17.51 (4.93–62.24)	0.91 (0.84–0.97)	19.28 (5.17–71.94)	0.99
CCP 3	4	—	—	—	—	—	—
Assay manufacturer							
Inova	8	0.17 (0.14–0.20)	0.99 (0.97–1.00)	15.19 (5.12–45.00)	0.84 (0.81–0.88)	18.01 (5.93–54.71)	0.80
Others	9	0.05 (0.02–0.11)	0.99 (0.98–1.00)	9.37 (1.78–49.19)	0.95 (0.91–1.00)	9.84 (1.80–53.88)	0.98

SN = sensitivity; SP = specificity; +LR = positive likelihood ratio; −LR = negative likelihood ratio; DOR = diagnostic odds ratio; AUC = area under curve; RF = rheumatoid factor; RF+ PA = RF-positive polyarthritis; RF− PA = RF-negative polyarthritis; SA = systemic-onset arthritis; OA = oligoarthritis; PsA = psoriatic arthritis; ERA = enthesitis-related arthritis; UA = undifferentiated arthritis; ORD = other rheumatic disease; HC = healthy control; CCP = cyclic citrullinated peptides; CCP1 = the first anti-cyclic citrullinated peptides autoantibody tests; CCP2 = the second anti-cyclic citrullinated peptides autoantibody tests; CCP3 = the third anti-cyclic citrullinated peptides autoantibody tests.

## Abbreviations

JIA: Juvenile idiopathic arthritis
LIAR: International League Against Rheumatism
RA: Rheumatoid arthritis
RF: Rheumatoid factor
RF− PA: Rheumatoid factor-negative polyarthritis
RF+ PA: Rheumatoid factor-positive polyarthritis
SA: Systemic-onset arthritis
OA: Oligoarthritis
PsA: Psoriatic arthritis
ERA: Enthesitis-related arthritis
UA: Undifferentiated arthritis
ANA antibody: Antinuclear antibody
AKA: Antikeratin
APF: Anti-perinuclear factor
anti-CCP: Anti-cyclic citrullinated peptide
ACR: American College of Rheumatology
QUADAS: Quality Assessment of Diagnostic Accuracy Studies
PLR: Positive likelihood ratio
NLR: Negative likelihood ratio
DOR: Diagnostic odds ratio
SROC: Summary receiver operating characteristic
TPR: True-positive rate
FPR: False-positive rate
HSROC: Hierarchical summary receiver operating characteristic.
